# Structural Characterization of an ACP from *Thermotoga maritima*: Insights into Hyperthermal Adaptation

**DOI:** 10.3390/ijms21072600

**Published:** 2020-04-09

**Authors:** Yeongjoon Lee, Ahjin Jang, Min-Cheol Jeong, Nuri Park, Jungwoo Park, Woo Cheol Lee, Chaejoon Cheong, Yangmee Kim

**Affiliations:** 1 Department of Bioscience and Biotechnology, Konkuk University, Seoul 05029, Korea; lyj7956@konkuk.ac.kr (Y.L.); ajin931017@konkuk.ac.kr (A.J.); boby8520@konkuk.ac.kr (M.-C.J.); snfl235@konkuk.ac.kr (N.P.); jhopark123@konkuk.ac.kr (J.P.); wclee3@konkuk.ac.kr (W.C.L.); 2 Magnetic Resonance Team, Korea Basic Science Institute, Ochang 28199, Korea; cheong@kbsi.re.kr

**Keywords:** Acyl carrier protein, NMR spectroscopy, structure, thermostability, *Thermotoga maritima*

## Abstract

*Thermotoga maritima*, a deep-branching hyperthermophilic bacterium, expresses an extraordinarily stable *Thermotoga maritima* acyl carrier protein (*Tm*-ACP) that functions as a carrier in the fatty acid synthesis system at near-boiling aqueous environments. Here, to understand the hyperthermal adaptation of *Tm*-ACP, we investigated the structure and dynamics of *Tm*-ACP by nuclear magnetic resonance (NMR) spectroscopy. The melting temperature of *Tm*-ACP (101.4 °C) far exceeds that of other ACPs, owing to extensive ionic interactions and tight hydrophobic packing. The D59 residue, which replaces Pro/Ser of other ACPs, mediates ionic clustering between helices III and IV. This creates a wide pocket entrance to facilitate the accommodation of long acyl chains required for hyperthermal adaptation of the *T. maritima* cell membrane. *Tm*-ACP is revealed to be the first ACP that harbor an amide proton hyperprotected against hydrogen/deuterium exchange for I15. The hydrophobic interactions mediated by I15 appear to be the key driving forces of the global folding process of *Tm*-ACP. Our findings provide insights into the structural basis of the hyperthermal adaptation of ACP, which might have allowed *T. maritima* to survive in hot ancient oceans.

## 1. Introduction

**Figure 1:**
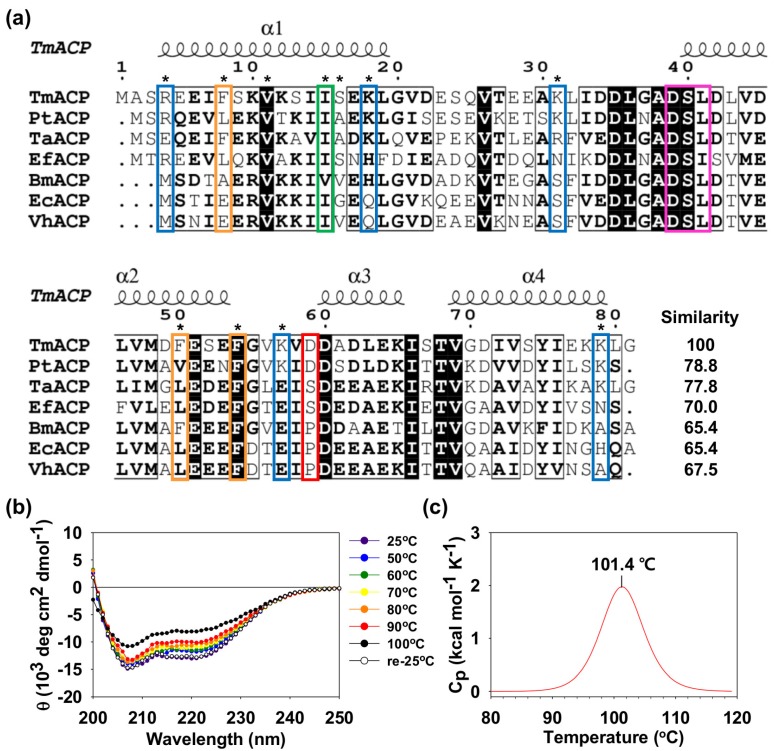
Sequence alignment of bacterial ACPs and the thermostability of *Thermotoga maritima* acyl carrier protein (*Tm*-ACP). (**a**) Sequence alignment of *Tm*-ACP with other bacterial ACPs, including hyperthermophilic *Pt*-ACP, thermophilic *Ta*-ACP, and mesophilic *Ef*-ACP, *Bm*-ACP, *Ec*-ACP, and *Vh*-ACP. 100% conserved residues are highlighted as black color, above 80% conservations are boxed by black lines. Mutated sites in this study are indicated by asterisks. All ACPs have conserved Ser residues that are linked to phosphopantetheine linkers (purple box). Unlike mesophilic proteins, hyperthermophilic ACPs have additional basic residues and nonconserved Asp residues, which are indicated by blue and red boxes, respectively. *Tm*-ACP has three Phe residues (orange boxes): one is highly conserved in all ACPs (F54) and two are rare (F8 and F50). I15 in *Tm*-ACP is nearly completely conserved among bacterial ACPs (green box). (**b**) Effect of temperature on the secondary structure of *Tm*-ACP investigated by circular dichroism (CD). (**c**) Melting temperature of wild-type *Tm*-ACP measured by differential scanning calorimetry (DSC).

Acyl carrier proteins (ACPs) are small (~9 kDa) acidic proteins that are essential for numerous biochemical pathways, including the biosynthesis of fatty acids, polyketides, lipopolysaccharides, lipoteichoic acids, rhizobial nodulation signaling factors, and pro-hemolysin toxins [1,2,3,4,5,6]. Fatty acid synthesis (FAS) is an essential process that produces fatty acids, which are important energy sources for cells and also serve as the building blocks of cell membranes and intracellular signaling substances [7]. As essential components in type II FAS systems [1,8], ACPs shuttle acyl intermediates in their hydrophobic pocket to facilitate interactions with various enzyme partners [9,10]. ACPs share highly conserved structures, including Asp-Ser-Leu (DSL) motifs at the N-terminus of helix II. A phosphopantetheine group is attached to the Ser residue (purple box in Figure 1a) by a phosphodiester linkage, and the free thiol group at the other end of the phosphopantetheine group can form a thioester bond with acyl groups [11,12,13,14]. The key structural features of various type II ACPs have been reported [15,16,17,18,19,20,21,22,23]. ACPs consist of four helical bundles connected by three loop regions, forming hydrophobic cavities to accommodate the growing acyl chains. ACPs are believed to be dynamic proteins, and their flexibilities are essential for their functions [10,24,25,26,27,28].

Protein structures have evolved to facilitate the adaption of the expressing organism to various environments. Proteins must maintain not only the correct structure but also the appropriate dynamic motion to accomplish their functions. Thermophilic proteins often contain extra salt bridges or tightly packed hydrophobic interactions, which help to maintain proper folding at high temperatures [29,30]. It is important to understand the mechanisms through which proteins balance structural stability and functional flexibility in such harsh environments. In this respect, *Thermotoga maritima* ACP (*Tm*-ACP) is a valuable target since *T. maritima* resides in extremely hot hydrothermal vents and is the only bacterium known to grow at temperatures up to 90 °C [31]. Its thermophilic cell membrane is highly enriched in saturated fatty acids with long chain lengths, which help maintain their liquid crystalline states at high temperatures [32,33]. Therefore, identification of the physicochemical properties of the hyperthermophilic ACP could provide important clues for understanding the functioning of the FAS system at extremely high temperatures. 

In order to gain insights into the thermophilic adaptation of the deep-branching bacterium *T. maritima* for surviving in hot aqueous environments, we investigated the structural factors contributing to the hyperthermostability of *Tm*-ACP. To this end, we investigated its melting temperature (T_m_) by differential scanning calorimetry (DSC) and its structural features by nuclear magnetic resonance (NMR) spectroscopy. Moreover, the thermodynamic parameters related to the folding of *Tm*-ACP were analyzed by NMR and circular dichroism (CD) experiments. These data will offer important clues for studying evolutionary strategies of the hyperthermophilic adaptation of *T. maritima* at the molecular level.

## 2. Results

### 2.1. Thermostability of Tm-ACP

Hyperthermophilic *Tm*-ACP shows high sequence similarities with other bacterial ACPs, ranging from 65.4% to 78.8% (Figure 1a). *Tm*-ACP has the conserved DSL motif (purple box in Figure 1a), where the Ser residue becomes connected to the phosphopantetheine linker. Despite this high sequence similarity, *Tm*-ACP also shows some unique sequence characteristics. Notably, although all bacterial ACPs have about 20 acidic residues, three thermophilic ACPs have double the number of positively charged residues compared to that found in mesophilic ACPs (Table 1). The nonconserved basic residues (blue boxes in Figure 1a) are located in various regions of *Tm*-ACP. The hyperthermophilic *Tm*-ACP and *Pt*-ACP both contain an Asp residue in the α_2_α_3_ loop which substitutes for the Pro or Ser of most mesophilic ACPs (red box in Figure 1a). *Tm*-ACP also has three Phe residues (orange boxes in Figure 1a), including the highly conserved F54 residue and two nonconserved residues, F8 and F50, resulting in increase of the structural stability of *Tm*-ACP.

The CD data demonstrated that *Tm*-ACP starts to denature at approximately 100 °C (Figure 1b). After cooling down the denatured sample to 25 °C, the α-helical structure was completely recovered, implying that the folding reaction of *Tm*-ACP is reversible. The specific T_m_ of *Tm*-ACP was measured as 101.4 °C by DSC (Figure 1c), which is the highest melting temperature for any ACP reported to date [15,18,34]. The reversibility was further confirmed by NMR spectroscopy, in which the ^1^H-^15^N heteronuclear single-quantum coherence (HSQC) spectrum of the *Tm*-ACP sample heated in boiling water for 15 min was identical to that of the native *Tm*-ACP (Figure S1a).

### 2.2. Tertiary Structure of Tm-ACP

**Figure 2:**
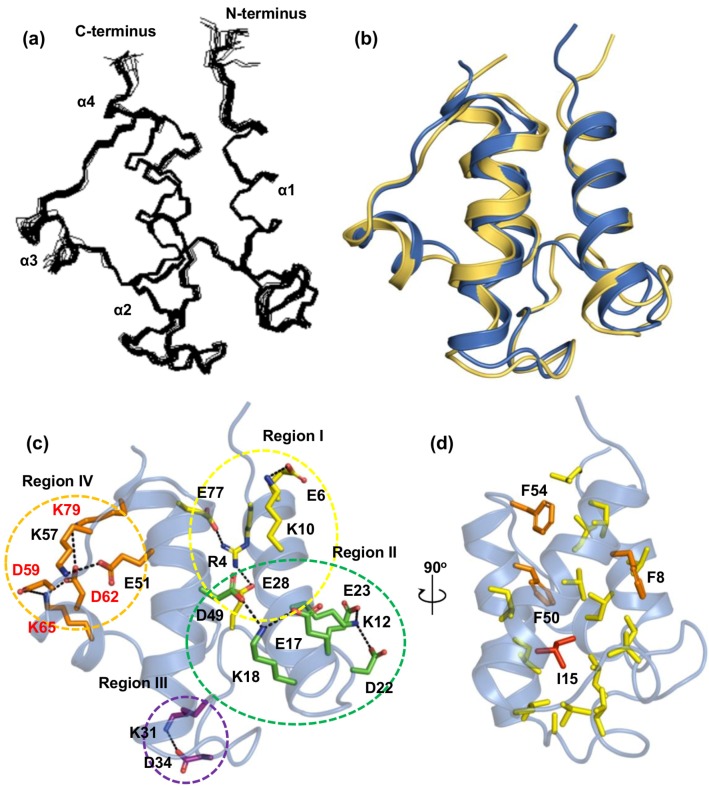
Structural features of holo *Tm*-ACP. (**a**) Superimposed backbone atoms (N, Ca, and C′) of the 20 lowest-energy solution structures of holo *Tm*-ACP. (**b**) Superimposition of two structures: the solution structure of holo *Tm*-ACP (blue) with the lowest energy and the crystal structure of apo *Tm*-ACP (yellow), where the backbone atoms of the helices are aligned (RMSD = 1.13 Å). (**c**) Electrostatic interactions in *Tm*-ACP divided into four different regions depicted in yellow, green, purple, and orange, respectively. Residues, which constitute the ionic cluster between helix III and IV (D59-K65-D62-K79), are labeled red. The positively charged nitrogen atoms in the guanidyl group of R4 and amino group of Lys residues form electrostatic interactions with the negatively charged oxygen atoms in the carboxylic groups of Asp and Glu residues. (**d**) Hydrophobic packing between three helices: I, II, and IV. The hydrophobic side chains of the packing residues are shown in yellow, except for the Phe residues (orange) and I15 (red).

To function as a carrier for acyl groups, an apo *Tm*-ACP should be converted to the holo form, where the phosphopantetheine linker is covalently connected to the side chain of S40 in the conserved DSL motif [12,13,14]. Both tertiary structures of the holo and apo forms of *Tm*-ACP were determined by NMR spectroscopy and X-ray crystallography, respectively (see associated statistics in Table 2; Table 3). In the final calculation of the solution structure of holo *Tm*-ACP, the well-superimposed 20 lowest-energy models with small root-mean square deviations (RMSDs; 0.3 and 0.7 Å) were obtained for all backbone and heavy atoms, respectively (Figure 2a). The overall completeness of assignment was 93% and the quality factor (Q) of residual dipolar coupling (RDC) data was calculated as 39.52% using Q = rms(RDC_measured_-RDC_calculated_)/rms(RDC_measured_) [36]. *Tm-*ACP consists of four α-helices (helix I (4–19), helix II (40–54), helix III (60–65), helix IV (69–80)) connected by a long α_1_α_2_ loop and two shorter loops. To accommodate the acyl chains, the hydrophobic cavities of ACPs can be expanded by protruding helix III outward [1,28,37]. Compared to the structure of holo *Ec*-ACP [38], the helix III of *Tm*-ACP was found to be protruded outward in both the apo and holo forms (Figure 2b and Figure S2). Hyperthermophilic *Tm*-ACP has nonconserved Glu and Lys residues (D59 and K79), which participate in an ionic cluster between helices III and IV (Figure 2c), forcing the outward protrusion of helix III.

The *Tm*-ACP structure has extensive electrostatic interactions in four different regions (Figure 2c). In region I (yellow), a nonconserved R4 residue forms ionic interactions with E28 and E77. In addition, E6 makes a salt bridge with K10. In region II (green), two ionic clusters, D22-K12-E23 and E17-K18-D49, further stabilize the C-terminal half of helix I, along with helix II and the long α_1_α_2_ loop. In region III (purple), a salt bridge between nonconserved residues K31 and D34 stabilizes a helical turn (L32–L36) located in the middle of the long α_1_α_2_ loop, thereby increasing the rigidity of this loop. Finally, in region IV (orange), the α_2_α_3_ loop and three helices, II, III and IV, are connected by extensive ionic interactions, including E51-K57 and D59-K65-D62-K79. This region is reported as the divalent cation-binding site and usually consists of many Glu residues in mesophilic ACPs [18,42]. The nonconserved K57 residue in hyperthermophilic *Tm*-ACP replaces these Glu residues, resulting in stabilization of the short α_2_α_3_ loop.

The interior of *Tm*-ACP is filled with numerous hydrophobic side chains, in which three Phe residues and I15 mediate the tight hydrophobic packing (Figure 2d). Located at the center of the packing, the hydrophobic side chain of I15 contact closely with those of 8 residues, V11 (2.1 Å), L19 (2.1 Å), V26 (3.1 Å), L32 (2.1 Å), L36 (2.4 Å), L46 (2.1 Å), F50 (2.4 Å), and V69 (3.1 Å). The shortest distances of each side chain from that of I15 were measured between the two closest protons, giving the average distance of 2.4 Å. *Tm*-ACP has a highly conserved aromatic F54 residue at the end of helix II along with additional Phe residues, F8 and F50 (Figure 1a). Similar to most ACP structures, F54 of *Tm*-ACP forms a hydrophobic triad with I7 and I76 at the top end of the hydrophobic cavity, fastening three helices: I, II, and IV. Nonconserved F8 at helix I forms hydrophobic contacts to V26 in the long α_1_α_2_ loop, and V69 and V73 in helix IV, further stabilizing the flexible α_1_α_2_ loop. Aromatic rings in F50 and F54 form additional hydrophobic interactions with other residues (I7, V11, I14, I15, L46, I72, I76, and L80) in the hydrophobic cavity. Nuclear Overhauser effects (NOEs) were observed for F50 with V11, I14, and I15, implying strong connections between helices I and II.

### 2.3. Key Residues Contributing to the Thermostability of Tm-ACP

To assess the roles of specific residues on the hyperthermal stability of *Tm-*ACP, the T_m_ of mutant *Tm*-ACPs were measured using DSC (Table 4). Mutations that replace residues involved in attractive electrostatic interactions by oppositely charged residues reduced the T_m_ by 4.2–23.5 °C. As R4 connects helix I, helix IV, and the α_1_α_2_ loop, the R4E mutant had much lower thermostability (T_m_ = 86.1 °C) compared to that of the wild-type protein. Replacement of K10 and K18 with Glu residues disrupted favorable electrostatic interactions, consequently reducing the T_m_ by 20.0 °C and 18.3 °C, respectively. In addition, since K12 stabilizes the α_1_α_2_ loop by forming salt bridges with D22 and E23, the replacement of K12 with Glu caused the most significant decrease in the thermostability of *Tm*-ACP, resulting in a T_m_ of 76.9 °C. Similarly, additional salt bridges formed by nonconserved Lys residues, K31 in the α_1_α_2_ loop and K57 in the α_2_α_3_ loop, appear to substantially contribute to the thermostability of *Tm*-ACP by stabilizing each loop. K31E and K57E mutants had reduced T_m_’s, 90.0 °C and 95.8 °C, respectively. Lastly, the K79E mutation caused the loss of the ionic cluster D59-K65-D62-K79, thereby reducing the thermostability of *Tm*-ACP by 10.6 °C. 

Previously, we have reported that a novel ACP from a heat-tolerant mesophile *Enterococcus faecalis* (*Ef*-ACP) has a high melting temperature of 78.8°C [18]. One of the key structural components that contribute to the high thermostability of *Ef*-ACP was revealed to be a nonconserved hydrogen bond between the side chain of S15 and the backbone of I20 in α_1_α_2_ loop. Similarly, S16 in the hyperthermophilic *Tm*-ACP also forms a hydrogen bond with the backbone of V21, and thus, further stabilizes the α_1_α_2_ loop along with the ionic interactions. As in the mesophilic *Ec*-ACP [38], the S16G mutant *Tm*-ACP lacking this hydrogen bond, resulting in a reduced T_m_ of 94.0 °C.

Hydrophobic interactions were also found to be important for the thermostability of *Tm*-ACP. We confirmed that three Phe residues contribute to its high thermostability. The F8A mutation resulted in destabilization of the α_1_α_2_ loop, thereby decreasing the thermostability by 6.3 °C. The F50A mutation within the hydrophobic cavity also lowered the T_m_ by 9.7 °C. In addition, as expected, the T_m_ of the F54A mutant decreased dramatically (81.8 °C), implying that the aromatic ring is crucial to the formation of the tight hydrophobic triad. Moreover, substitutions of hydrophobic core residues such as V11, I15, I72, and V73 with Ala also markedly dropped the T_m_, revealing their essential roles in the tight hydrophobic packing of *Tm*-ACP. In particular, the T_m_ of I15A dropped significantly (84.6 °C). 

### 2.4. Hydrogen/Deuterium (H/D) Exchange Experiments 

**Figure 3:**
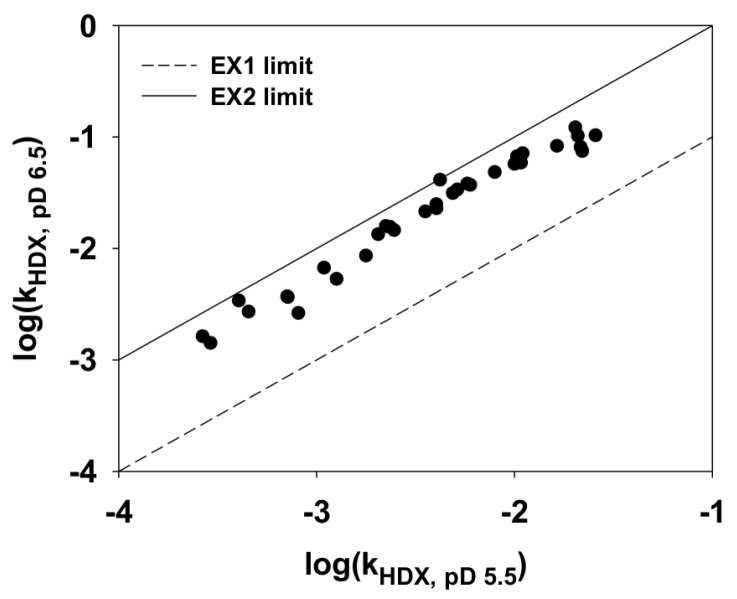
Log-log plot of the exchange rate constants (k_HDX_) of amide protons in holo *Tm*-ACP measured at pD 5.5 and pD 6.5 at 25 °C. All amide protons that had computable k_HDX_ values were included. The solid line represents the EX2 limit, log(k_HDX_, _pD 6.5_) = log(k_HDX, pD 5.5_) + 1, and the dotted line represents the EX1 limit, log(k_HDX_, _pD 6.5_) = log(k_HDX, pD 5.5_).

**Figure 4:**
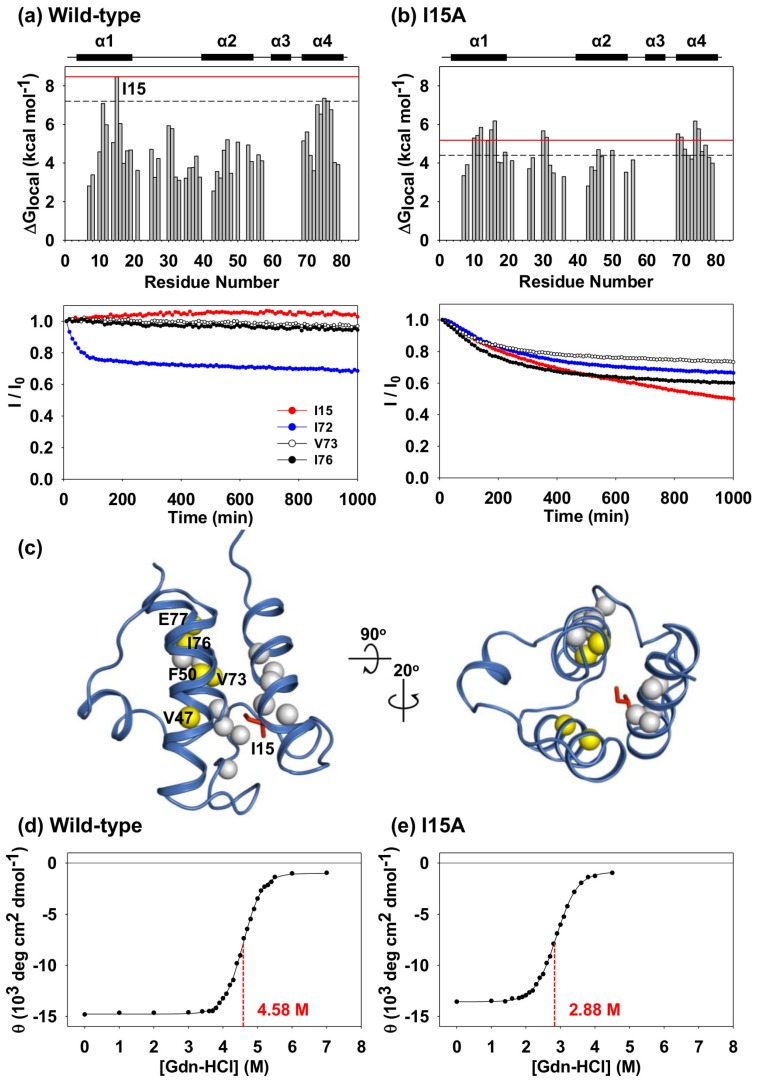
H/D exchange and Gdn-HCl induced unfolding experiments of *Tm*-ACP. Free energies of local unfolding (ΔG_local_) of amide protons in (**a**) wild-type and (**b**) I15A mutant holo *Tm*-ACPs from H/D exchange experiments. Red solid lines in the free energy of local unfolding plots indicate the respective free energy of global unfolding (ΔG_global_) at 25 °C. Black dashed lines indicate the lower limit of the global unfolding regime, [0.85]ΔG_global_. Decay curves of normalized peak intensities for the core hydrophobic packing residues are indicated as a function of time after the addition of D_2_O. (**c**) The amide protons of sixteen residues with ΔG_local_ > 5kcal/mol were shown as spheres on the structure of *Tm*-ACP. As the replacement of A15 for I15 in the I15A mutant weakened the overall hydrophobic packing, two residues at the helix II (V47 and F50) and three residues at the helix IV (V73, I76, and E77), showed decreased ΔG_local_ values. The amide protons of those five residues were denoted in yellow. Gdn-HCl induced global unfolding of (**d**) wild-type and (**e**) I15A mutant holo *Tm*-ACPs was observed by monitoring the mean residue ellipticity at 222 nm with different concentrations of Gdn-HCl. Before the measurements, all samples were kept at 25 °C for 12 h to achieve complete equilibration of denaturation processes.

To explore the protection of amide protons and the local unfolding of hyperthermophilic *Tm*-ACP, the free energies of local unfolding (ΔG_local_) in *Tm*-ACP were determined by H/D exchange experiments. For slowly exchanged amide protons, whose peaks are shown on the first ^1^H-^15^N HSQC spectrum after a 10 min exchange, the exchange rate constants (k_HDX_) were determined from the decay of each peak height over a 1000 min exchange [44]. In cases in which the amide protons are exchanged by an EX2 mechanism [45], k_HDX_ approximates to K_unfold_ × k_rc_ in the base-catalyzed regime of pD 5–7 [46], where K_unfold_ is the equilibrium constant of the local unfolding reaction and k_rc_ is the pD-dependent exchange rate for the random coil conformation. Because k_HDX_ is determined by K_unfold_ and k_rc_, its value depends highly on pD. Within the range of pD 5–7, the log(k_HDX_) of amide protons increases by one unit as pD increases by one unit [44]. In contrast, k_HDX_ in an EX1 mechanism is only characterized by the local unfolding rate constant (k_HDX_ = k_unfold_), and pD has no effect on k_HDX_. To confirm whether the exchange reaction of amide protons in *Tm*-ACP followed the EX2 mechanism, the pD dependences of k_HDX_ of amide protons were compared at pD 5.5 and pD 6.5. As shown in Figure 3, all slowly exchanged amide protons in *Tm*-ACP behaved in accordance with the EX2 limit, implying that their exchanges were generally based on the EX2 mechanism. Because the equilibrium constant of the local unfolding reaction (K_unfold_) could be approximated by k_HDX_ / k_rc_, their free energies of local unfolding (ΔG_local_) could be determined by Equation (1) [46],

ΔG_local_ = − RT ln(K_unfold_) (1)

where R is the universal gas constant, and T is the experimental temperature (25 °C). As expected, the amide protons of *Tm*-ACP were highly protected against the H/D exchange reaction, except for those in regions near helix III (Figure 4a). Ten minutes after the addition of D_2_O, 46 amide peaks survived. The peaks of eight residues, V11, K12, I15, V69, I72, V73, I76, and E77, remained even after 1 month, indicating that these local regions required large energies to be unfolded. Surprisingly, the peak intensity of I15 was maintained at a nearly constant level for 1 month, making it impossible to determine its k_HDX_ and ΔG_local_ from the H/D exchange experiment. This suggested that the amide proton of I15 could only be exposed to the solvent if the tertiary structure of the protein was completely denatured under a scenario of global unfolding. 

To verify the importance of I15, we mutated I15 into an Ala residue. The H/D exchange experiment showed that weakened hydrophobic packing due to the I15A mutation reduced the free energies for the local unfolding of *Tm*-ACP (Figure 4b), demonstrating a critical role of this highly protected residue in the folding process. Compared with wild-type *Tm*-ACP, the I15A mutant protein had fewer amide peaks remaining on the first spectrum. The amide peaks of eight residues (Q25, D35, G37, A38, D39, M48, E53, and K57) in I15A completely disappeared after 10 min, which were retained for several hours in the wild-type protein. Furthermore, the amide protons of the core hydrophobic residues also showed faster exchange than those in the wild-type protein. Only the peaks of seven residues (V11, A15, V69, I72, V73, I76, and E77) remained after 1000 min. Similar to wild-type *Tm*-ACP, these peaks could be observed even after 1 month, but with much weaker intensities. 

For the wild-type protein, there were sixteen residues, V11, K12, I14, I15, S16, A30, K31, V47, F50, V69, G70, V73, S74, Y75, I76, and E77, which had large ΔG_local_ (> 5kcal/mol) since their amide protons were located near the compact hydrophobic packing mediated by I15 (Figure 4c). Compared to this, replacement of A15 for I15 in the I15A mutant weakened the overall hydrophobic packing. As a result, two residues at the helix II (V47 and F50) and three residues at the helix IV (V73, I76, and E77) had decreased ΔG_local_ values (yellow spheres). This implies that the size and hydrophobicity of the side chain of I15 is important for maintaining the tight packing in the structure of *Tm*-ACP.

### 2.5. Chemical Denaturation of Tm-ACP

To measure the stability of *Tm*-ACP upon chemical denaturation and confirm the importance of I15 in protein folding, we determined the free energies of global unfolding (ΔG_global_) of wild-type *Tm*-ACP and the I15A mutant using far-UV CD experiments [47]. The CD data were analyzed using a reversible two-state model for the native (N)-to-denatured (U) equilibrium and the linear extrapolation model shown in Equation (2) [47,48,49,50],

ΔG_global, [Gdn-HCl]_ = ΔG_global_ − m[Gdn-HCl] (2)

where ΔG_global, [Gdn-HCl]_ is the free energy of global unfolding at a certain Gdn-HCl concentration ([Gdn-HCl]), ΔG_global_ is the free energy of global unfolding at a zero-denaturant concentration, and m is the slope of the fitted plot. ΔG_global, [Gdn-HCl]_ was calculated by fitting the data to equations (3) and (4).

K_global, [Gdn-HCl]_ = f_U_ / f_N_ = (1 − f_N_) / f_N_ (3)

G_global, [Gdn-HCl]_ = –RT ln(K_global, [Gdn-HCl]_) (4)

The fraction of the native state (f_N_) was obtained from the resulting mean residue ellipticity values detected at 222 nm, and that of the unfolded state (f_U_) was calculated as the sum of all fractions (f_N_ + f_U_), which is always equal to one for a reversible two-state equilibrium. The equilibrium constant of the global unfolding reaction at a certain Gdn-HCl concentration (K_global, [Gdn-HCl]_) was then calculated using Equation (3) [48,49]. Finally, ΔG_global_ was determined by rearranging Equation (2) to Equation (5) and substituting the Gdn-HCl concentration into the measured mid-point concentration ([Gdn-HCl]_1/2_) [46,47,50].

ΔG_global_ = m[Gdn-HCl]_1/2_ (5)

Because the free energy required for local unfolding cannot exceed that required for global unfolding, we indirectly deduced the k_HDX_ and ΔG_local_ values of hyperprotected I15 by approximating ΔG_local_ to ΔG_global_. Notably, the hyperthermostable *Tm*-ACP was also found to be exceptionally stable against Gdn-HCl-induced denaturation with an unusually high mid-point concentration ([Gdn-HCl]_1/2_) of 4.58 M (Figure 4d), resulting in a ΔG_global_ value of 8.47 kcal mol-1. Similar to the results for thermal denaturation, the I15A mutation destabilized the structure of *Tm*-ACP, substantially lowering [Gdn-HCl]_1/2_ and ΔG_global_ to 2.88 M and 5.18 kcal mol^−^^1^, respectively (Figure 4e). This implies that the hydrophobic side chain of I15 is a key factor for the global folding process of *Tm*-ACP.

## 3. Discussion 

**Figure 5:**
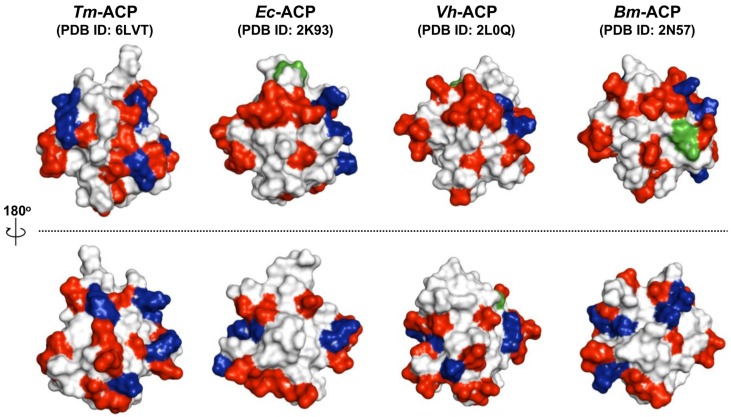
Distributions of the surface charges in bacterial ACPs. All coordinates were obtained from Protein Data Bank (PDB) [15,23,38]. For all ACPs, negatively charged Glu and Asp residues are indicated in red and positively charged Arg and Lys residues are shown in blue. His residues (green) can also provide a positive charge in a physiological pH range. Only the hyperthermophilic *Tm*-ACP has extensive ionic interactions on its surface.

Hyperthermophilic proteins are more rigid than their mesophilic counterparts, and this structural rigidity is commonly considered as a prerequisite for high thermostability [51,52,53,54]. Salt bridges have been proposed to play a crucial role in increasing the rigidity and thermostability of hyperthermophilic proteins [29,30]. In addition to ionic interactions, hydrophobic interactions have also been reported to provide additional stabilization to the structures of thermophilic proteins [55,56]. In this study, we found that the ACP from a hyperthermophile, *T. maritima*, had extensive noncovalent interactions and thus an extremely thermostable structure with a melting temperature of 101.4°C. Compared to mesophilic ACPs, the hyperthermophilic *Tm*-ACP has additional positive charges on its surface (Figure 1a, Figure 5). These positive charges not only neutralize the destructive repulsions between nearby negative charges, but also mediate extensive ionic interactions at the exterior surface of *Tm*-ACP, stabilizing the overall structure. Moreover, *Tm*-ACP is the first ACP shown to harbor hyperprotected amide protons, requiring large free energies to be unfolded. This suggested that in addition to the compact hydrophobic interactions with I15, the distinct stabilization factors near the amide proton of I15 provided extraordinarily high rigidity at this local region, leading to hyperprotection.

Local unfolding events with ΔG_local_ close to ΔG_global_ may be identical to the global unfolding process [46]. To identify the amide sites that are unfolded only by the global unfolding process, each ΔG_local_ from H/D exchange experiments was compared with the ΔG_global_ value obtained from chemical denaturation experiments. For accurate comparison, chemical denaturation experiments were performed under the same buffer and temperature conditions that were used in the H/D exchange experiments. Similar to the results of Laity et al. [46], we defined a ΔG_local_ higher than 85% of ΔG_global_ (ΔG_local_ > [0.85]ΔG_global_) as identical to ΔG_global_, considering the uncertainties of the measurements. The amide sites of I15 in helix I and two residues in helix IV, Y75 and I76, were found in the global unfolding regime (ΔG_local_ > [0.85]ΔG_global_) for the wild-type *Tm*-ACP at 25 °C. This implies that the hydrophobic side chain of I15 mediated tight hydrophobic packing between helix I and IV and that this packing may act as the last energy barrier of the global unfolding process of *Tm*-ACP. Because the folding reaction of *Tm*-ACP is reversible, the hydrophobic packing mediated by I15 may drive tertiary folding at very early steps by promoting hydrophobic packing among helix I, helix IV, and the α_1_α_2_ loop.

As shown in Figure S3, the I15A mutation caused large chemical shift perturbation in most of residues, implying that the replacement of A15 for I15 in the I15A mutant made the hydrophobic packing looser, and thereby, might cause significant change in the conformation of *Tm*-ACP. Although the amide proton of A15 in the I15A mutant was no longer as well-protected as I15 in wild-type *Tm*-ACP, it still played a role in the global unfolding process given the dramatic decrease in ΔG_global_. In addition, the ^1^H-^15^N HSQC spectrum of the heat-treated I15A mutant also completely recovered (Figure S1b), implying that the mutation did not affect the reversibility of the folding reaction of *Tm*-ACP. These results indicate that the small hydrophobic side chain of A15 was also able to mediate the hydrophobic packing essential for the folding, but significantly lowered the energy barrier toward the unfolded state. Therefore, the proper size of the hydrophobic side chain at this conserved Ile site is critical for tight hydrophobic packing to promote the folded states of ACPs.

**Figure 6:**
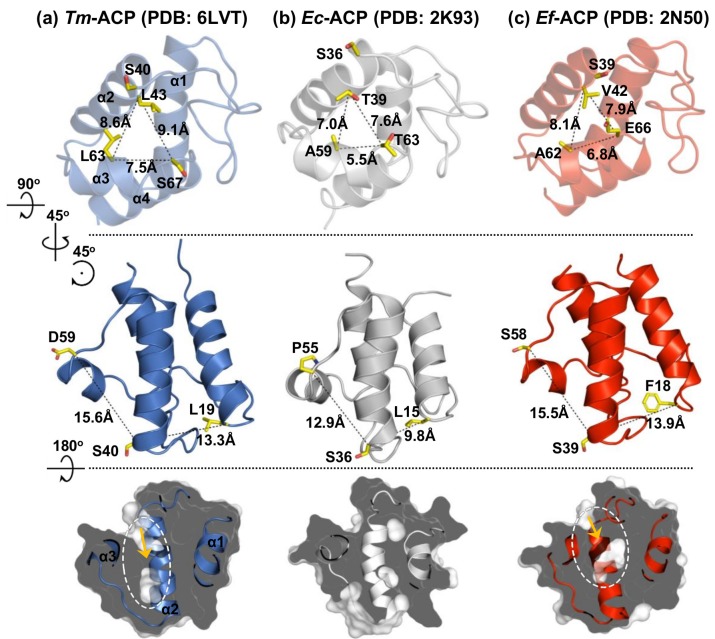
Structural comparison of bacterial ACPs. The top row depicts the size of each pocket entrance of (**a**) *Tm*-ACP (PDB ID: 6LVT), (**b**) *Ec*-ACP (PDB ID: 2K93) [38], and (**c**) *Ef*-ACP (PDB ID: 2N50) [18]. The size of the entrances was indicated as the distances between the three outermost residues that form each entrance. The middle row shows the distances between three helices, I, II, and III, of each protein. The bottom row displays the detectable cavities within the ACPs.

Bacterial cell membranes mostly consist of fatty acyl chains and have their own phase-transition temperatures resulting from variations in the lengths, degrees of saturation, and compositions of the acyl chains [33]. The hyperthermophile *T. maritima* possesses numerous long fatty acids, which comprise the thermostable cell membrane of the organism [32]. Therefore, *Tm*-ACP should be able to accommodate longer acyl chains than other mesophilic ACPs. In the crystal structure of mesophilic *Escherichia coli* ACP (*Ec*-ACP), the T39, A59, and T63 residues were revealed as the three outermost residues that form the entrance and interact with the prosthetic group [57]. The distances among these residues in holo *Ec*-ACP were 7.0 Å, 5.5 Å, and 7.6 Å, respectively (Figure 6a). For hyperthermophilic *Tm*-ACP, the distances between the Cα atoms of the corresponding residues, L43, L63, and S67, were increased to 8.6 Å, 7.5 Å, and 9.1 Å, respectively (Figure 6b), forming a much larger entrance. This would facilitate the entrance of long acyl chains into the hydrophobic pocket of *Tm*-ACP.

An atypical ACP (*Ef*-ACP) was found to be expressed as an auxiliary ACP that acts as a carrier for de novo fatty acid synthesis. After incorporating fatty acids from the host, these exogenous fatty acids are loaded to the atypical *Ef*-ACP and shuttled to the canonical FAS enzymes [58]. Since the exogenous acyl chains are frequently found to be long and unsaturated like oleic acid [58], *Ef*-ACP should have a large hydrophobic pocket to accommodate those long acyl cargos. In this respect, helices I and III of *Ef*-ACP were revealed to be protruded away from helix II, making an expanded space within the hydrophobic pocket [18]. The distances between three helices, I, II, and III, are greater in *Ef*-ACP than those in *Ec*-ACP (Figure 6c). Similarly, the hyperthermophilic *Tm*-ACP also showed increased distances, thereby forming an expanded binding pocket. Therefore, along with the wide entrance, the expansion of the pocket seems to be crucial for the function of *Tm*-ACP in shuttling long acyl chains for the thermal adaptation of the *T. maritima* cell membrane.

## 4. Materials and Methods 

### 4.1. Cloning, Expression, Isotopic Enrichment, and Purification

The *acpP* gene of *T. maritima* MSB8 was first cloned into the multi-cloning site of pET-11a vector by using two restriction enzymes, NdeI (catatg) and BamHI (ggatcc). The recombinant pET-11a vector was transformed into *E. coli* BL21 (DE3) [18]. To express isotope-labeled proteins, pre-cultured recombinant cells were inoculated into 500 mL of M9 minimal medium containing 50 mg/L ampicillin and isotope-enriched ^15^NH_4_Cl and ^13^C-glucose (Cambridge Isotope Laboratories, Andover, MA, USA). After the optical density at a wavelength of 600 nm (OD_600_) reached to 0.8–1.0, 1mM isopropyl β-D-1-thiogalactopyranoside (IPTG) was added to induce the overexpression of *Tm*-ACP. The medium was placed at 37 °C and incubated for 6 hours. Purification of *Tm*-ACP was achieved by using its physicochemical properties, the surface charge (HiTrap Q FF and Resource Q, GE Healthcare Bio-Sciences, Uppsala, Sweden) and the molecular size (Superdex 75 16/600, GE Healthcare Life Sciences, Uppsala, Sweden) [18]. Usually, 5–10 mg of protein was yielded from 1 L of culture. After purification as the apo protein, *Tm*-ACP was converted to its holo form by using holo-ACP synthase from *E. coli* (*Ec*-AcpS) and coenzyme A (CoA) at 25 °C for 12 h [18]. The 25 mM Tris-HCl (pH 8) buffer with 20 mM MgCl_2_ was used as a reaction buffer.

### 4.2. Site-Directed Mutagenesis

All mutation processes for *Tm*-ACP mutants were performed by polymerase chain reaction (PCR) amplification using various mutagenetic primer pairs (Appendix A Table A1). 10 fM of the pET-11a vector which contains the wild-type *acpP* gene of *T. maritima* MSB8 was used as a template. Each primer was used at a final concentration of 0.2 μM. The building blocks (dATP, dGTP, dTTP, and dCTP; 0.2 mM each) and 2 μM of nPfu-Forte DNA polymerase were added. After 30 cycles of denaturation (94 °C, 1 min), annealing (60 °C, 1 min), and elongation (72 °C, 5 min), the amplified vectors were transformed into *E. coli* BL21 (DE3). All mutant proteins were expressed and purified just the same as the wild-type protein.

### 4.3. NMR Experiments and Assignments

NMR spectroscopy experiments were performed using the Bruker Avance 700, 800 and 900 MHz spectrometers at the Korea Basic Science Institute (Ochang, Korea). 0.4–0.5 mM of the ACP samples was prepared in 330 μL of 9:1 (*v/v*) H_2_O/D_2_O 25 mM 2-(N-morpholino)ethanesulfonic acid (MES) buffer (pH 6.1) containing 5 mM CaCl_2_ and 5 mM dithiothreitol (DTT). 2,2-dimethyl-2-silapentane-5-sulfonate (DSS) was used as an internal chemical shift reference. 0.02% of NaN_3_ was added as an antiseptic. Triple resonance spectra of HNCO, HNCACB, and CBCA(CO)NH experiments were acquired to assign the resonances of spins within the backbones of *Tm*-ACP. For side chain assignment, CC(CO)NH, HBHA(CO)NH, H(CCO)NH, and HCCH-TOCSY spectra were obtained. The assignments were confirmed by ^1^H-^15^N-^1^H and ^1^H-^13^C-^1^H NOESY-HSQC spectra [18,59,60,61,62]. All NMR spectra were processed with NMRPipe [63] and analyzed with NMRFAM-Sparky [64]. Residual dipolar coupling (RDC) constants between two spins in backbone amide N-H bonds were determined by comparing spatially anisotropic dipolar couplings in IPAP-HSQC spectra of solution and gel phase *Tm*-ACP sample. The gel phase sample was prepared by dissolving the solution sample in a radially compressed polyacrylamide gel [18,62,65,66,67].

### 4.4. Solution Structure Calculation

Nuclear Overhauser effect (NOE) assignments were carried out using NMRFAM-Sparky [64], and the 3D structure of holo *Tm*-ACP was determined using Xplor-NIH-based calculations in the PONDEROSA-C/S package [39]. Thereafter, 20 lowest-energy structures were determined. All angle and distance violations, of the best 20 structures were analyzed and refined using PONDEROSA-Analyzer [40]. The final 20 lowest-energy structures were evaluated using PSVS [41]. Figures for the protein structures were generated using PyMOL (http://www.pymol.org). Final coordinates and NOE constraints have been deposited in the Protein Data Bank (PDB) under the accession number 6LVT (BMRB ID: 36242).

### 4.5. Hydrogen/Deuterium Exchange Experiments

The ^15^N-labeled *Tm*-ACP samples (0.5 mM) prepared for the NMR experiment were lyophilized for the H/D exchange experiment. D_2_O (100%) was added right before the experiments, and the successive HSQC data were collected every 10 min using two scans and time domains of 1000/256 for 1000 min at 25 °C. Additional HSQC spectra were acquired after 3, 7, 14, 21, and 28 days to monitor the surviving cross peaks. Each exchange rate constant (k_HDX_) of amide protons was determined from the decay of peak height over time. For amide protons, which are exchanged by an EX2 mechanism [45], the exchange rate (k_HDX_) approximated by k_HDX_ = K_unfold_ × k_rc_ in the base-catalyzed regime of pD 5–7, where K_unfold_ is the equilibrium constant of the local unfolding reaction (K_unfold_ = k_unfold_ / k_fold_) and k_rc_ is the exchange rate for the random coil conformation [46,68]. Thus, K_unfold_ approximates to k_HDX_ / k_rc_. To acquire k_rc_ for individual amide protons in the protein, k_rc_ values for poly-dl-alanine at 293K (20 °C) as a function of pD [69] were first corrected by taking into consideration the inductive and steric effects of neighboring side chains [44]. Then, k_rc_ values at 25 °C were calculated using the equation k_HDX,T_ = k_HDX,293K_ Exp(-E_a_(1/T – 1/293)), where the temperature T is 298 K (25 °C) and E_a_ is the activation energy [44]. The local unfolding energies (ΔG_local_) were also determined from K_unfold_ [46].

### 4.6. Circular Dichroism Experiment

Secondary structure of *Tm*-ACP at various temperatures was assessed by Far-UV CD experiment using a J810 spectropolarimeter (Jasco, Tokyo, Japan). Thirty μM of the protein was dissolved in 25 mM MES buffer (pH 6.1) containing 5 mM CaCl_2_ and 5 mM dithiothreitol (DTT) and placed in a cuvette with a 1 mm path length. CD spectra were measured from 200 to 250 nm at 0.1 nm intervals. Temperature was increased gradually from 25 to 100 °C. The thermal denaturation of the protein was observed by monitoring the change in the mean residue ellipticities (θ) at 222 nm wavelength. θ was calculated as described previously [70,71].

### 4.7. Chemical Denaturation Experiment

The chemical denaturant-induced global unfolding of wild-type and mutant *Tm*-ACPs was also investigated using Far-UV CD experiments. In brief, 30 μM of the proteins were dissolved in 25 mM MES buffer (pH 6.1) containing 5 mM CaCl_2_ and 5 mM DTT, and then different concentrations of Gdn-HCl were added to the protein solutions. After incubation at 25 °C for 12 h, complete equilibrium was achieved for all samples. The CD spectra of the samples were measured from 215 to 250 nm at 0.1-nm intervals. The CD data were analyzed using a reversible two-state model for the native (N)-to-denatured (U) equilibrium and the linear extrapolation model (Equation (2)) [47,48,49,50]. ΔG_global, [Gdn-HCl]_ was than calculated by Equations (3) and (4) [48,49]. Finally, ΔG_global_ was determined by using equation (5) [46,47,50].

### 4.8. Differential Scanning Calorimetry

The melting temperatures of wild-type and mutant *Tm*-ACPs were measured by DSC using a NanoDSC system (TA instruments, New Castle, DE, USA). The protein samples were prepared at concentrations of 2 mg/mL in 20 mM potassium phosphate buffer (pH 7.0). After degassing for 10 min, the reference buffer and the protein samples were equilibrated at 50 °C for 10 min. The thermograms were recorded as the temperature was increased at a rate of 1°C/min from 50 °C to 120 °C. During the measurements, the pressure was kept constant at 3 atm to prevent the phase transition of the solvent. After polynomial baseline corrections and two-state scaled curve fittings, individual component peaks were resolved from the complex profiles.

### 4.9. X-ray Crystallography

Apo *Tm*-ACP was crystallized by 0.2 M zinc acetate dihydrate with 18–24% polyethylene glycol (PEG) 3350 precipitant at 20 °C. All crystals were harvested in the same buffer with 20% ethylene glycol as a cryoprotectant and stored in liquid nitrogen until data collection. X-ray diffraction data were collected at the beamlines 5C and 7A of the Pohang Accelerator Laboratory (Pohang, Korea). Crystals were maintained at −173 °C during data collection to prevent radiation damage. Diffraction images were integrated and scaled using the HKL2000 program suite [72]. The tertiary structure of apo *Tm*-ACP was determined by molecular replacement using the coordinates of *Aquifex aeolicus* ACP (PDB ID: 2EHS) as a search model. The initial structure was built by PHENIX [73] and modified using the WinCoot.[74] Average B-factors of the protein, water, and ligands were calculated by MOLEMAN2 [43]. Final coordinates of apo *Tm*-ACP were deposited in Protein Data Bank (PDB) under accession number 6LVU.

## 5. Conclusions

Hyperthermophilic *Tm*-ACP maintains its structure even at extremely high temperatures to function as an acyl carrier in the FAS system of *T. maritima*. Therefore, it is valuable to study the structural properties of this protein for understanding the adaptation strategies of proteins toward the extremely hot environments. Here, we provided the first NMR structure of *Tm*-ACP and demonstrated that extensive electrostatic interactions and enhanced hydrophobic packings cumulatively stabilize the structure of *Tm*-ACP, allowing it to perform its carrier function in the FAS system at extremely high temperatures. An ionic cluster between helices III and IV mediated by the nonconserved D59 residue forces the outward protrusion of helix III, resulting in a wide entrance of the hydrophobic cavity to facilitate the accommodation of long acyl chains required for thermal adaptation of the cell membrane of *T. maritima*. Moreover, *Tm*-ACP is the first ACP proven to harbor a hyperprotected amide proton for I15, which is also identified to be a key residue involved in global folding of the protein. It mediates hydrophobic interactions between helix I, II, and IV, and thus, facilitates the folding process of *Tm*-ACP. Our new insights into the structural properties of the hyperthermophilic *Tm*-ACP may provide a molecular understanding of the adaptational strategies employed by the primitive hyperthermophile to withstand the extreme temperatures associated with hot ancient marine environments.

## Figures and Tables

**Table 1:** Comparison of isoelectric points and the number of charged residues of bacterial ACPs.

	*Tm*-ACP ^1^	*Pt*-ACP ^2^	*Ta*-ACP ^3^	*Ef*-ACP ^4^	*Bm*-ACP ^5^	*Ec*-ACP ^6^	*Vh*-ACP ^7^
pI ^8^	4.13	4.28	4.29	3.87	3.97	3.98	3.79
Acidic (Glu/Asp)	21 (10/11)	19 (8/11)	21 (14/7)	21 (11/10)	20 (9/11)	20 (14/6)	22 (14/8)
Basic (Arg/Lys)	9 (1/8)	10 (1/9)	10 (2/8)	5 (1/4)	6 (1/5)	5 (1/4)	6 (1/5)

^1^ Thermotoga maritima ACP; ^2^ Pseudothermotoga thermarum ACP; ^3^ Thermus aquaticus ACP; ^4^ Enterococcus faecalis ACP; ^5^ Brucella melitenis ACP; ^6^ Escherichia coli ACP; ^7^ Vibrio harveyi ACP; ^8^ The isoelectric points (pI) of bacterial ACPs were calculated by ProtParam tool (https://web.expasy.org/protparam) [35].

**Table 2:** Statistics of solution structure of holo *Tm*-ACP. The 20 lowest-energy structures were determined by NMR spectroscopy.

Restraints ^1^	
Total	1108
Conformationally restricting distance constraints	
Short Range ((i– j) <= 1)	301
Medium Range (1 < (i – j) ≤ 5)	266
Long Range ((i – j) > 5)	196
Dihedral angle constraints	
Phi	77
Psi	77
Hydrogen-bond constraints	114
Residual dipolar coupling (RDC) constraints	77
Xplor-NIH pseudo-potential energy (kJ/mol) ^2^	3251
**Average Rmsd to the Mean Coordinates (Å) ^3^**	
Backbone atoms (all / ordered residues ^4^)	0.3/0.2
Heavy atoms (all / ordered residues ^4^)	0.7/0.6
**Ramachandran Plot Summary from PROCHECK (%) ^3^**	
Most favored regions	96.4
Allowed regions	3.6
Disallowed regions	0.0
**Average Number of Violations Per Conformer ^2^**	
Distance constraint violations (> 0.2 Å)	0
Angle constraint violations (> 10°)	0

^1^ The solution structure of holo *Tm*-ACP was calculated using Xplor-NIH-based calculation in PONDEROSA-C/S [39]. ^2^ Xplor-NIH pseudo-potential energy and all violations of the 20 best structures were analyzed using PONDEROSA-Analyzer [40]. ^3^ The final 20 lowest-energy structures were evaluated using PSVS (Protein Structure Validation Software) [41]. ^4^ Ordered residues: S3-L80.

**Table 3:** Statistics of crystal structure of apo *Tm*-ACP.

**Data collection**	
Space group	P2_1_2_1_2_1_
Unit-cell	
a, b, c (Å)	23.83, 61.95, 95.72
α, β, γ (^o^)	90, 90, 90
Resolution (Å)	28.37–2.29
Unique reflections	49,345
Redundancy	7.3
Completeness for range (%)	98.2
Mean I/σ(I)	26.65 (at 2.29 Å)
R_merge_ (%) ^1^	8.1
**Refinement ^2^**	
No. of reflections (overall)	6797
No. of reflections (test set)	671
R-factor ^3^	0.2217(0.2104 at 2.29 Å)
R-free ^4^	0.2841(0.2762 at 2.29 Å)
RMSZ ^5^ / RMSD	
Bond lengths (Å)	0.42/0.008
Bond angles (^o^)	0.63/1.086
Wilson B-factor (Å^2^) ^6^	19.5
**Ramachandran Plot Summary from PROCHECK (%) ^2^**	
Most favored regions	97.2
Allowed regions	2.8
Disallowed regions	0.0
**Average B-factors (Å^2^) ^7^**	
Protein	27.3
Water	27.9
Ligand	40.0

^1^ R_merge_=Σ_hkl_Σ_i_(I_i_–I_m_)/Σ_hkl_Σ_i_I_m_, where I_i_ is the i-th measurement and I_m_ is the weighted mean intensity of the reflection. ^2^ Evaluated by PSVS (Protein Structure Validation Software) [41]. ^3^ R=Σ|(|F_obs_|-|F_calc_|)|/Σ(|F_obs_|). ^4^ R-free was calculated as the R-factor for 5% of the data randomly omitted from the refinement. ^5^ RMSZ is the root-mean-square of all Z scores of the bond lengths (or angles). The Z score for a bond length (or angle) is the number of standard deviations the observed value is removed from the expected value (|Z| > 5 is considered an outlier worth inspection). ^6^ An estimate of the overall B-value of the structure, calculated from the diffraction data. ^7^ The mean B-value calculated over the modelled atoms by using MOLEMAN2 [43].

**Table 4:** Melting temperatures (T_m_) of mutant *Tm*-ACPs ^1.^

Related Interaction	Phenotype	T_m_ (°C)
	Wild-type	100.4
Electrostatic	R4E	86.1
	K10E	80.4
	K12E	76.9
	K18E	82.1
	K31E	90.0
	K57E	95.8
	K79E	89.8
Hydrogen bond	S16G	94.0
Hydrophobic	F8A	94.1
	F50A	90.7
	F54A	81.8
	V11A	91.6
	I15A	84.6
	I72A	90.1
	V73A	89.7

^1^ As the contribution of the prosthetic group to the thermostability of *Tm*-ACP was found to be negligible, all T_m_ values of mutants were measured and compared in their apo forms.

## Supplementary Materials

Supplementary materials can be found at https://www.mdpi.com/1422-0067/21/7/2600/s1, Figure S1: Reversibility of the thermal denaturation of (a) the wild-type and (b) the I15A mutant holo *Tm*-ACPs was confirmed by comparing the ^1^H-^15^N HSQC spectra of each protein before (blue) and after (red) heat-treatments. The annealing was performed by heating the native sample in a boiling water bath for 15 min and cooling down to room temperature, Figure S2: Superimposition of three structures: the solution structure of holo *Tm*-ACP (blue, PDB ID: 6LVT) with the lowest energy, the crystal structure of apo *Tm*-ACP (yellow, PDB ID: 6LVU) and the solution structure of holo *Ec*-ACP (grey, PDB ID: 2k93) [38]. The red arrows indicate the protrusion of helix III of *Tm*-ACP compared to that of *Ec*-ACP, Figure S3: ^1^H-^15^N HSQC spectral overlay of the wild-type (black) and the I15A mutant (red) holo *Tm*-ACPs. The chemical shift perturbation of the eight residues, V11, L19, V26, L32, L36, L46, F50, and V69, whose sidechains contact closely with that of I15, were indicated as blue arrows.

## Abbreviations

ACP	Acyl carrier protein
ACPS	Holo-ACP synthase
*Bm*	*Brucella melitenis*
CD	Circular dichroism
CoA	Coenzyme A
DSC	Differential scanning calorimetry
DSL	Asp-Ser-Leu
DSS	2,2-Dimethyl-2-silapentane-5-sulfonate
DTT	Dithiothreitol
*Ec*	*Escherichia coli*
*Ef*	*Enterococcus faecalis*
FAS	Fatty acid synthesis
H/D	Hydrogen/deuterium
HSQC	Heteronuclear single quantum coherence spectroscopy
IPTG	Isopropyl β-D-1-thiogalactopyranoside
NOE	Nuclear Overhauser effect
OD	Optical density
PCR	polymerase chain reaction
PDB	Protein data bank
PEG	Polyethylene glycol
*Pt*	*Pseudothermotoga thermarum*
RDC	Residual dipolar coupling
RMSD	Root mean square deviation
RMSZ	Root mean square of Z-scores
*Ta*	*Thermus aquaticus*
*Tm*	*Thermotoga maritima*
*Vh*	*Vibrio harveyi*

## Appendix A

**Table A1:** Primers for the site-directed mutagenesis of *Tm*-ACP.

Mutant	Direction	Primer Sequence
R4E	forward	5′-gaa gga gat ata cat atg gcc agt gaa gaa gaa att ttt tct aaa gtg aaa tc-3′
	reverse	5′-gat ttc act tta gaa aaa att tct tct tca ctg gcc ata tgt ata tct cct tc-3′
K10E	forward	5′-cgg gaa gaa att ttt tct gaa gtg aaa tcc atc atc tc-3′
	reverse	5′-gag atg atg gat ttc act tca gaa aaa att tct tcc cg-3′
K12E	forward	5′-gaa att ttt tct aaa gtg gaa tcc atc atc tct gaa-3′
	reverse	5′-ttc aga gat gat gga ttc cac ttt aga aaa aat ttc-3′
K18E	forward	5′-cca tca tct ctg aag aat tgg ggg tcg atg-3′
	reverse	5′-cat cga ccc cca att ctt cag aga tga tgg-3′
K31E	forward	5′-gtg acg gaa gag gcc gaa ttg att gat gat ctg gga g-3′
	reverse	5′-ctc cca gat cat caa tca att cgg cct ctt ccg tca c-3′
K57E	forward	5′-gtg agt tcg gcg ttg aag tcg atg acg ccg-3′
	reverse	5′-cgg cgt cat cga ctt caa cgc cga act cac-3′
K79E	forward	5′-gtc agc tac att gaa aaa gaa tta ggc tag gga tcc ggc tgc-3′
	reverse	5′-gca gcc gga tcc cta gcc taa ttc ttt ttc aat gta gct gac-3′
S16G	forward	5′-cta aag tga aat cca tca tcg gtg aaa aat tgg ggg tcg atg aat c -3′
	reverse	5′-gat tca tcg acc ccc aat ttt tca ccg atg atg gat ttc act tta g-3′
F8A	forward	5′-gcc agt cgg gaa gaa att gcg tct aaa gtg aaa tcc tag-3′
	reverse	5′-cta gga ttt cac ttt aga cgc aat ttc ttc ccg act ggc-3′
F50A	forward	5′-gac ctg gta atg gac gcg gag agt gag ttc gg-3′
	reverse	5′-ccg aac tca ctc tcc gcg tcc att acc agg tc-3′
F54A	forward	5′-gga ctt tga gag tga ggc ggg cgt taa agt cg′a tg-3′
	reverse	5′-cat cga ctt taa cgc ccg cct cac tct caa agt cc-3′
V11A	forward	5′-cgg gaa gaa att ttt tct aaa gcg aaa tcc atc atc tct gaa aaa ttg g-3′
	reverse	5′-cca att ttt cag aga tga tgg att tcg ctt tag aaa aaa ttt ctt ccc g-3′
I15A	forward	5′-gaa att ttt tct aaa gtg aaa tcc atc gcc tct gaa aaa ttg ggg gtc g-3′
	reverse	5′-cga ccc cca att ttt cag agg cga tgg att tca ctt tag aaa aaa ttt c-3′
I72A	forward	5′-gaa aat ctc tac tgt ggg cga cgc cgt cag cta cat tga aaa aaa att ag-3′
	reverse	5′-cta att ttt ttt caa tgt agc tga cgg cgt cgc cca cag tag aga ttt tc-3′
V73A	forward	5′-cta ctg tgg gcg aca ttg cca gct aca ttg aaa aaa aat tag-3′
	reverse	5′-cta att ttt ttt caa tgt agc tgg caa tgt cgc cca cag tag-3′

## References

[1] Chan D.I., Vogel H.J. (2010). Current understanding of fatty acid biosynthesis and the acyl carrier protein. Biochem. J..

[2] Lim J., Kong R., Murugan E., Ho C.L., Liang Z.X., Yang D. (2011). Solution structures of the acyl carrier protein domain from the highly reducing type I iterative polyketide synthase CalE8. PLoS ONE.

[3] Masoudi A., Raetz C.R.H., Zhou P., Pemble C.W. (2014). Chasing acyl carrier protein through a catalytic cycle of lipid A production. Nature.

[4] Heaton M.P., Neuhaus F.C. (1994). Role of the D-alanyl carrier protein in the biosynthesis of D-alanyl-lipoteichoic acid. J. Bacteriol..

[5] Geiger O., Spaink H.P., Kennedy E.P. (1991). Isolation of the *Rhizobium leguminosarum* NodF nodulation protein: NodF carries a 4'-phosphopantetheine prosthetic group. J. Bacteriol..

[6] Issartel J.P., Koronakis V., Hughes C. (1991). Activation of *Escherichia coli* prohaemolysin to the mature toxin by acyl carrier protein-dependent fatty acylation. Nature.

[7] Wakil S.J., Stoops J.K., Joshi V.C. (1983). Fatty acid synthesis and its regulation. Annu. Rev. Biochem..

[8] Crosby J., Crump M.P. (2012). The structural role of the carrier protein--active controller or passive carrier. Nat. Prod. Rep..

[9] Chan D.I., Stockner T., Tieleman D.P., Vogel H.J. (2008). Molecular Dynamics Simulations of the Apo-, Holo-, and Acyl-forms of *Escherichia coli* Acyl Carrier Protein. J. Biol. Chem..

[10] Nguyen C., Haushalter R.W., Lee D.J., Markwick P.R.L., Bruegger J., Caldara-Festin G., Finzel K., Jackson D.R., Ishikawa F., O’Dowd B. (2014). Trapping the dynamic acyl carrier protein in fatty acid biosynthesis. Nature.

[11] Wu B.N., Zhang Y.M., Jie Z., Rock C.O. (2004). Key residues responsible for acyl carrier protein (ACP) and beta-ketoacyl-acyl carrier protein reductase (FabG) interaction. Biophys. J..

[12] Flugel R.S., Hwangbo Y., Lambalot R.H., Cronan J.E., Walsh C.T. (2000). Holo-(acyl carrier protein) synthase and phosphopantetheinyl transfer in *Escherichia coli*. J. Biol. Chem..

[13] Sztain T., Patel A., Lee D.J., Davis T.D., McCammon J.A., Burkart M.D. (2019). Modifying the Thioester Linkage Affects the Structure of the Acyl Carrier Protein. Angew. Chem. Int. Ed. Engl..

[14] Johnson M.N., Londergan C.H., Charkoudian L.K. (2014). Probing the phosphopantetheine arm conformations of acyl carrier proteins using vibrational spectroscopy. J. Am. Chem. Soc..

[15] Chan D.I., Chu B.C., Lau C.K., Hunter H.N., Byers D.M., Vogel H.J. (2010). NMR solution structure and biophysical characterization of *Vibrio harveyi* acyl carrier protein A75H: Effects of divalent metal ions. J. Biol. Chem..

[16] Crump M.P., Crosby J., Dempsey C.E., Parkinson J.A., Murray M., Hopwood D.A., Simpson T.J. (1997). Solution structure of the actinorhodin polyketide synthase acyl carrier protein from *Streptomyces coelicolor* A3(2). Biochemistry.

[17] Holak T.A., Kearsley S.K., Kim Y., Prestegard J.H. (1988). Three-dimensional structure of acyl carrier protein determined by NMR pseudoenergy and distance geometry calculations. Biochemistry.

[18] Park Y.G., Jung M.C., Song H., Jeong K.W., Bang E., Hwang G.S., Kim Y. (2016). Novel Structural Components Contribute to the High Thermal Stability of Acyl Carrier Protein from *Enterococcus faecalis*. J. Biol. Chem..

[19] Sharma A.K., Sharma S.K., Surolia A., Surolia N., Sarma S.P. (2006). Solution structures of conformationally equilibrium forms of holo-acyl carrier protein (*Pf*ACP) from *Plasmodium falciparum* provides insight into the mechanism of activation of ACPs. Biochemistry.

[20] Wong H.C., Liu G.H., Zhang Y.M., Rock C.O., Zheng J. (2002). The solution structure of acyl carrier protein from *Mycobacterium tuberculosis*. J. Biol. Chem..

[21] Xu G.Y., Tam A., Lin L., Hixon J., Fritz C.C., Powers R. (2001). Solution structure of *B. subtilis* acyl carrier protein. Structure.

[22] Barnwal R.P., Van Voorhis W.C., Varani G. (2011). NMR structure of an acyl-carrier protein from *Borrelia burgdorferi*. Acta Crystallogr. Sect. F Struct. Biol. Cryst. Commun..

[23] Barnwal R.P., Kaur M., Heckert A., Gartia J., Varani G. (2020). Comparative structure, dynamics and evolution of acyl-carrier proteins from *Borrelia burgdorferi*, *Brucella melitensis* and *Rickettsia prowazekii*. Biochem. J..

[24] Kim Y., Kovrigin E.L., Eletr Z. (2006). NMR studies of *Escherichia coli* acyl carrier protein: Dynamic and structural differences of the apo- and holo-forms. Biochem. Biophys. Res. Commun..

[25] Lim J., Xiao T., Fan J., Yang D. (2014). An off-pathway folding intermediate of an acyl carrier protein domain coexists with the folded and unfolded states under native conditions. Angew. Chem. Int. Ed. Engl..

[26] Colizzi F., Masetti M., Recanatini M., Cavalli A. (2016). Atomic-Level Characterization of the Chain-Flipping Mechanism in Fatty-Acids Biosynthesis. J. Phys. Chem. Lett..

[27] Zhou Y., Yang D. (2017). Equilibrium folding dynamics of meACP in water, heavy water, and low concentration of urea. Sci. Rep..

[28] Arya R., Sharma B., Dhembla C., Pal R.K., Patel A.K., Sundd M., Ghosh B., Makde R.D., Kundu S. (2019). A conformational switch from a closed apo- to an open holo-form equips the acyl carrier protein for acyl chain accommodation. Biochim. Biophys. Acta Proteins Proteom..

[29] Brininger C., Spradlin S., Cobani L., Evilia C. (2018). The more adaptive to change, the more likely you are to survive: Protein adaptation in extremophiles. Semin. Cell Dev. Biol..

[30] Hait S., Mallik S., Basu S., Kundu S. (2019). Finding the generalized molecular principles of protein thermal stability. Proteins.

[31] Huber R., Langworthy T.A., König H., Thomm M., Woese C.R., Sleytr U.B., Stetter K.O. (1986). *Thermotoga maritima* sp. nov. represents a new genus of unique extremely thermophilic eubacteria growing up to 90°C. Arch. Microbiol..

[32] Carballeira N.M., Reyes M., Sostre A., Huang H.S., Verhagen M.F.J.M., Adams M.W.W. (1997). , Unusual fatty acid compositions of the hyperthermophilic archaeon *Pyrococcus furiosus* and the bacterium *Thermotoga maritima*. J. Bacteriol..

[33] Koga Y. (2012). Thermal adaptation of the archaeal and bacterial lipid membranes. Archaea.

[34] Horvath L.A., Sturtevant J.M., Prestegard J.H. (1994). Kinetics and thermodynamics of thermal denaturation in acyl carrier protein. Protein Sci..

[35] Wilkins M.R., Gasteiger E., Bairoch A., Sanchez J.C., Williams K.L., Appel R.D., Hochstrasser D.F. (1999). Protein identification and analysis tools in the ExPASy server. Methods Mol. Biol..

[36] Cornilescu G., Marquardt J.L., Ottiger M., Bax A. (1998). Validation of protein structure from anisotropic carbonyl chemical shifts in a dilute liquid crystalline phase. J. Am. Chem. Soc..

[37] Ramelot T.A., Rossi P., Forouhar F., Lee H.W., Yang Y., Ni S., Unser S., Lew S., Seetharaman J., Xiao R. (2012). Structure of a specialized acyl carrier protein essential for lipid A biosynthesis with very long-chain fatty acids in open and closed conformations. Biochemistry.

[38] Wu B.N., Zhang Y.M., Rock C.O., Zheng J.J. (2009). Structural modification of acyl carrier protein by butyryl group. Protein Sci..

[39] Lee W., Stark J.L., Markley J.L. (2014). PONDEROSA-C/S: Client-server based software package for automated protein 3D structure determination. J. Biomol. NMR.

[40] Lee W., Cornilescu G., Dashti H., Eghbalnia H.R., Tonelli M., Westler W.M., Butcher S.E., Henzler-Wildman K.A., Markley J.L. (2016). Integrative NMR for biomolecular research. J. Biomol. NMR.

[41] Bhattacharya A., Tejero R., Montelione G.T. (2007). Evaluating protein structures determined by structural genomics consortia. Proteins.

[42] Frederick A.F., Kay L.E., Prestegard J.H. (1988). Location of divalent ion sites in acyl carrier protein using relaxation perturbed 2D NMR. FEBS Lett..

[43] Kleywegt G.J. (1997). Validation of protein models from Calpha coordinates alone. J. Mol. Biol..

[44] Bai Y., Milne J.S., Mayne L., Englander S.W. (1993). Primary structure effects on peptide group hydrogen exchange. Proteins.

[45] Englander S.W. (2000). Protein folding intermediates and pathways studied by hydrogen exchange. Annu. Rev. Biophys. Biomol. Struct..

[46] Laity J.H., Montelione G.T., Scheraga H.A. (1999). Comparison of local and global stability of an analogue of a disulfide-folding intermediate with those of the wild-type protein in bovine pancreatic ribonuclease A: Identification of specific regions of stable structure along the oxidative folding pathway. Biochemistry.

[47] Santoro M.M., Bolen D.W. (1992). A test of the linear extrapolation of unfolding free energy changes over an extended denaturant concentration range. Biochemistry.

[48] Pace C.N. (1986). Determination and analysis of urea and guanidine hydrochloride denaturation curves. Methods Enzymol..

[49] Schellman J.A. (1987). The thermodynamic stability of proteins. Annu. Rev. Biophys. Biophys. Chem..

[50] Padmanabhan S., Laurents D.V., Fernandez A.M., Elias-Arnanz M., Ruiz-Sanz J., Mateo P.L., Rico M., Filimonov V.V. (1999). Thermodynamic analysis of the structural stability of phage 434 Cro protein. Biochemistry.

[51] Zavodszky P., Kardos J., Svingor A., Petsko G.A. (1998). Adjustment of conformational flexibility is a key event in the thermal adaptation of proteins. Proc. Natl. Acad. Sci. USA.

[52] Jaenicke R., Bohm G. (1998). The stability of proteins in extreme environments. Curr. Opin. Struct. Biol..

[53] Gershenson A., Schauerte J.A., Giver L., Arnold F.H. (2000). Tryptophan phosphorescence study of enzyme flexibility and unfolding in laboratory-evolved thermostable esterases. Biochemistry.

[54] Vieille C., Zeikus G.J. (2001). Hyperthermophilic enzymes: Sources, uses, and molecular mechanisms for thermostability. Microbiol. Mol. Biol. Rev..

[55] Fang X., Cui Q., Tong Y., Feng Y., Shan L., Huang L., Wang J. (2008). A stabilizing alpha/beta-hydrophobic core greatly contributes to hyperthermostability of archaeal [P62A]Ssh10b. Biochemistry.

[56] Tych K.M., Batchelor M., Hoffmann T., Wilson M.C., Hughes M.L., Paci E., Brockwell D.J., Dougan L. (2016). Differential Effects of Hydrophobic Core Packing Residues for Thermodynamic and Mechanical Stability of a Hyperthermophilic Protein. Langmuir.

[57] Roujeinikova A., Simon W.J., Gilroy J., Rice D.W., Rafferty J.B., Slabas A.R. (2007). Structural studies of fatty acyl-(acyl carrier protein) thioesters reveal a hydrophobic binding cavity that can expand to fit longer substrates. J. Mol. Biol..

[58] Zhu L., Zou Q., Cao X., Cronan J.E. (2019). *Enterococcus faecalis* Encodes an Atypical Auxiliary Acyl Carrier Protein Required for Efficient Regulation of Fatty Acid Synthesis by Exogenous Fatty Acids. mBio.

[59] Lee J., Jeong K.W., Jin B., Ryu K.S., Kim E.H., Ahn J.H., Kim Y. (2013). Structural and dynamic features of cold-shock proteins of *Listeria monocytogenes*, a psychrophilic bacterium. Biochemistry.

[60] Jeong K.W., Kang D.I., Lee E., Shin A., Jin B., Park Y.G., Lee C.K., Kim E.H., Jeon Y.H., Kim E.E. (2014). Structure and backbone dynamics of vanadate-bound PRL-3: Comparison of 15N nuclear magnetic resonance relaxation profiles of free and vanadate-bound PRL-3. Biochemistry.

[61] Jin B., Jeong K.W., Kim Y. (2014). Structure and flexibility of the thermophilic cold-shock protein of *Thermus aquaticus*. Biochem. Biophys. Res. Commun..

[62] Lee Y., Kwak C., Jeong K.W., Durai P., Ryu K.S., Kim E.H., Cheong C., Ahn H.C., Kim H.J., Kim Y. (2018). Tyr51: Key Determinant of the Low Thermostability of the *Colwellia psychrerythraea* Cold-Shock Protein. Biochemistry.

[63] Delaglio F., Grzesiek S., Vuister G.W., Zhu G., Pfeifer J., Bax A. (1995). NMRPipe: A multidimensional spectral processing system based on UNIX pipes. J. Biomol. NMR.

[64] Lee W., Tonelli M., Markley J.L. (2015). NMRFAM-SPARKY: Enhanced software for biomolecular NMR spectroscopy. Bioinformatics.

[65] Chou J.J., Gaemers S., Howder B., Louis J.M., Bax A. (2001). A simple apparatus for generating stretched polyacrylamide gels, yielding uniform alignment of proteins and detergent micelles. J. Biomol. NMR.

[66] Sass H.J., Musco G., Stahl S.J., Wingfield P.T., Grzesiek S. (2000). Solution NMR of proteins within polyacrylamide gels: Diffusional properties and residual alignment by mechanical stress or embedding of oriented purple membranes. J. Biomol. NMR.

[67] Cordier F., Dingley A.J., Grzesiek S. (1999). A doublet-separated sensitivity-enhanced HSQC for the determination of scalar and dipolar one-bond J-couplings. J. Biomol. NMR.

[68] Gangadhara B.N., Laine J.M., Kathuria S.V., Massi F., Matthews C.R. (2013). Clusters of Branched Aliphatic Side Chains Serve As Cores of Stability in the Native State of the HisF TIM Barrel Protein. J. Mol. Biol..

[69] Molday R.S., Englander S.W., Kallen R.G. (1972). Primary structure effects on peptide group hydrogen exchange. Biochemistry.

[70] Kelly S.M., Jess T.J., Price N.C. (2005). How to study proteins by circular dichroism. Biochim. Biophys. Acta.

[71] Greenfield N.J. (2006). Using circular dichroism spectra to estimate protein secondary structure. Nat. Protoc..

[72] Otwinowski Z., Minor W. (1997). Processing of X-ray diffraction data collected in oscillation mode. Methods Enzymol..

[73] Adams P.D., Afonine P.V., Bunkoczi G., Chen V.B., Davis I.W., Echols N., Headd J.J., Hung L.W., Kapral G.J., Grosse-Kunstleve R.W. (2010). PHENIX: A comprehensive Python-based system for macromolecular structure solution. Acta Crystallogr. D Biol. Crystallogr..

[74] Emsley P., Cowtan K. (2004). Coot: Model-building tools for molecular graphics. Acta Crystallogr. D Biol. Crystallogr..

