# Supplementary material for: Structural Characterization of an ACP from Thermotoga maritima: Insights into Hyperthermal Adaptation
Source: Int J Mol Sci. 2020 Apr 9;21(7):2600. doi: 10.3390/ijms21072600 (PMC7178038; doi:10.3390/ijms21072600)
Supplement: Supplementary file 1 [file ijms-21-02600-s001.pdf]

International Journal of
Molecular Sciences

MDPI

Supplementary Information for an Article Titled:

Structural Characterization of an ACP from *Thermotoga maritima*: Insights into Hyperthermal Adaptation

Yeongjoon Lee [1], Ahjin Jang [1], Min-Cheol Jeong [1], Nuri Park [1], Jungwoo Park [1], Woo Cheol Lee [1], Chaejoon Cheong [2] and Yangmee Kim [1,*]

[1] Department of Bioscience and Biotechnology, Konkuk University, Seoul 05029, Korea; lyj7956@konkuk.ac.kr (Y.L.); ajin931017@konkuk.ac.kr (A.J.); boby8520@konkuk.ac.kr (M.-C.J.); snfl235@konkuk.ac.kr (N.P.); jhopark123@konkuk.ac.kr (J.P.); wclee3@konkuk.ac.kr (W.C.L.)

[2] Magnetic Resonance Team, Korea Basic Science Institute, Ochang 28199, Chungcheongbuk-do, Korea; cheong@kbsi.re.kr

* Correspondence: ymkim@konkuk.ac.kr; Tel.: +82-2-450-3421

Received: 12 March 2020; Accepted: 7 April 2020; Published: 9 April 2020

© 2020 by the authors. Submitted for possible open access publication under the terms and conditions of the Creative Commons Attribution (CC BY) license (http://creativecommons.org/licenses/by/4.0/).

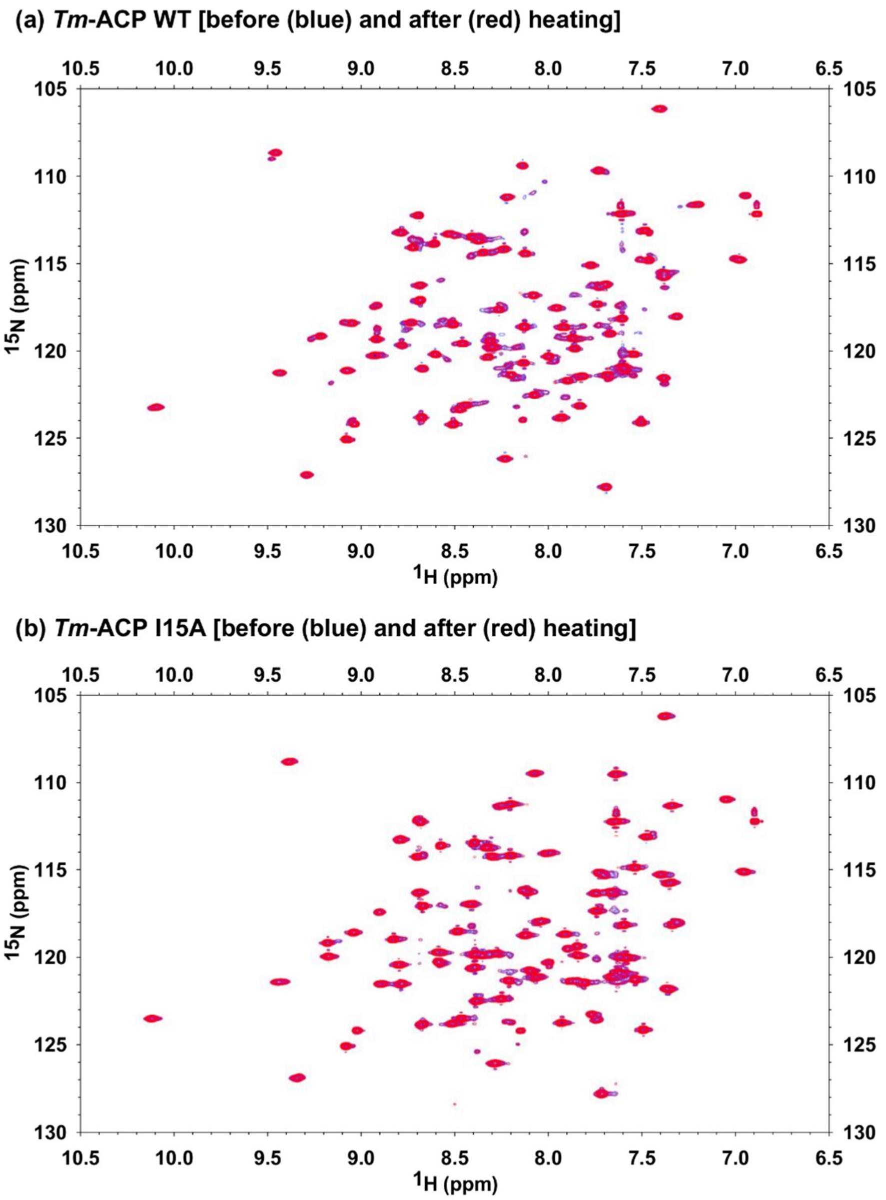

Figure S1. Reversibility of the thermal denaturation of (a) the wild-type and (b) the I15A mutant holo *Tm*-ACPs was confirmed by comparing the ^{1}H-^{15}N HSQC spectra of each protein before (blue) and after (red) heat-treatments. The annealing was performed by heating the native sample in a boiling water bath for 15 min and cooling down to room temperature.

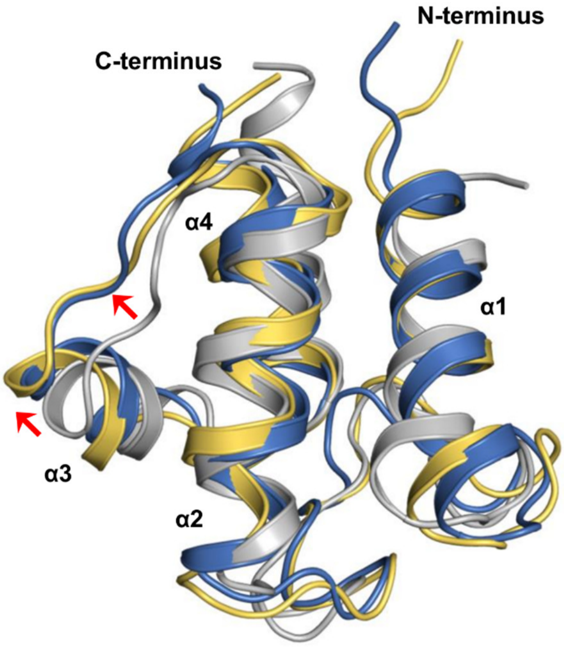

Figure S2. Superimposition of three structures: the solution structure of holo *Tm*-ACP (blue, PDB ID: 6LVT) with the lowest energy, the crystal structure of apo *Tm*-ACP (yellow, PDB ID: 6LVU) and the solution structure of holo *Ec*-ACP (grey, PDB ID: 2k93) [38]. The red arrows indicate the protrusion of helix III of *Tm*-ACP compared to that of *Ec*-ACP.

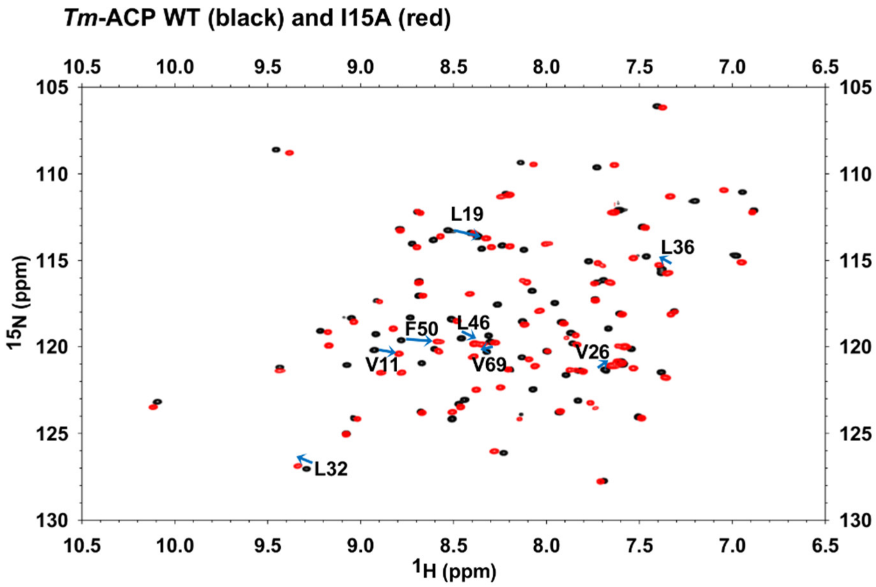

Figure S3. ^{1}H-^{15}N HSQC spectral overlay of the wild-type (black) and the I15A mutant (red) holo *Tm*-ACPs. The chemical shift perturbation of the eight residues, V11, L19, V26, L32, L36, L46, F50, and V69, whose sidechains contact closely with that of I15, were indicated as blue arrows.
